# Union of Medical Societies in New York

**Published:** 1902-01

**Authors:** 


**A Monthly Review of Medicine and Surgery.**

*EDITOR:*

WILLIAM WARREN POTTER, M. D.

All communications, whether of a literary or business nature, books for review and exchanges should be addressed to the editor: 284 FRANKLIN STREET, BUFFALO, N. Y.

VOL. XLI.—LVII. JANUARY, 1902. No. 6.

### Union of Medical Societies in New York.

AT THE regular monthly meeting of the Medical Society of the County of New York, held Monday, December 2, 1901, Dr. Frank Van Fleet, the newly inaugurated president, in his address recommended that some steps be taken toward resumption of relationship with the American Medical Association.

In pursuance of this suggestion Dr. D. B. St. John Roosa offered the following:

*Resolved*, That the president of the Medical Society of the County of New York appoint a committee of five, provided a similar committee be named by the New York County Medical Association, to confer with that body with reference to a union of the two organisations; and that this committee be requested to report to the society, at a stated meeting in January, 1902, or sooner, in order that this society may, if desirable make a recommendation to the Medical Society of the State of New York at its annual meeting.

The foundation is therefore being laid for a restoration of good fellowship between the profession of the state of New York and the National Medical Association. We had intended to make extended comment on the subject but some of our contemporaries have already discussed it quite fully. Among them *American Medicine* in its issue of December 14, 1901. This representative journal has presented the case so completely and in so much better light than we could, that we venture to present the entire article:

MEDICAL REUNION FOR NEW YORK.

At the last regular meeting of the Medical Society ot the County of New York, the air was full of suggestions as to the necessity for reunion and harmony among the members of the profession in New York State. The New York County Society is perhaps the oldest regular medical organisation in the country. To its initiative is due the New York State Medical Society, which was largely instrumental in the formation of the American Medical Association. Unfortunately, for some fifteen years the Medical Society of the County of New York and the State Society have not been in affiliation with the National Medical Association. The cause of the retirement of the New York State Society and its dependent organisations from the National Association was a difference of opinion as to the application of certain principles of the code of ethics.

At the November meeting of the New York County Society, the retiring president, Dr. George R. Fowler, spoke of the eminent desirability of reconciliation between the two regular medical organisations at present existent in New York County and State. The newly elected president, Dr. Frank Van Fleet, dwelt on the benefits of reunion. These would accrue not only to New York, but also to the whole country, for if the medical influence of the Empire State was concentrated instead of divided as at present, the medical profession would be a much more important factor for the accomplishment of state and national medical purposes. The new president considers that the present medical organisations should be ready to make any sacrifices consistent with honor to bring about professional reunion.

Dr. St. John Roosa then moved that a committee of five be appointed to confer with a similar committee from the County Medical Association for the purpose of arranging, if possible, the details of some method by which harmony and union could be brought about between the two county, and eventually also the state, organisations. The report of this committee it is hoped will be in shape to present some definite scheme of union at the next annual meeting of the Medical Society of the state, which takes place at Albany at the beginning of February, 1902.

This professional disunion has been the one serious rift in the lute of medical harmony in this country. Let us hope then that the first actual step toward reunion has been taken by the recent action of the County Society. There is not a member of the American Medical Association who has its interests at heart who would not welcome the news that the New York split was a thing of the past. Serious principles are supposed by many to be involved in the disunion. If we may trust the last president of the American Medical Association disunion was due rather to misconception than to absolute differences on principles. In his presidential address at St. Paul, Dr. Reed said:

This action (the opprobrious excommunication for the time being of the entire profession of the great Empire State) viewed impartially after the lapse of twenty years becomes the more extraordinary when it is observed that similar action was never taken with regard to Massachusetts, or Rhode Island or Mississippi, the societies of neither of which had ever adopted prescribed rules of conduct; nor with regard to California or Illinois or Colorado, each of which had by overt act if not by open declaration so far as this rule is concerned at least taken an equally nonconformist position.

Dr. Reed goes on to say that today there are forty-eight states or territorial licensing boards, most of them being composed of both the regular and sectarian schools of practice. The laws of the different states are of varying efficiency, the one procured by the Medical Society of the State of New York "at the price of yet maintained excommunication from this body, standing today as the model of excellence for the entire country." Yet it was practically for the assertion of its right to encourage such legislation that New York was voted out of the American Medical Association.

With regard to the merits of the question as to the code of ethics, Dr. Reed said further:

The committee on reorganisation (of the American Medical Association) under the restrictions of the resolution creating it has very properly left undisturbed the existing rules of conduct. These, if construed to have a fundamental importance and if vigorously enforced as they now stand, would disintegrate the association in a single day. This reason and others already given confirm me in the conviction that such rules should be either amended or abrogated. Or if reaffirmed it should be by general resolution endorsing their underlying principles, but disclaiming the present applicability of their details.

After such frank official declarations it would seem that there was ample room in the generous bosom of the mother association for all her sons from New York. We sincerely hope that the committee who meet for the preliminary discussion of the subject of union will realize fully the eminent desirability of complete medical union. Surely no trivial objections or personal feeling should be allowed to delay the fulfilment of so important a desideratum. There is a responsibility in the matter, not alone to local organisations but to the American Medical Association and to the profession of the whole country.

The foregoing is a comprehensive and just review of the situation, by an impartial observer looking on from a distance, yet, nevertheless, competent to estimate the disgrace of the present chaotic condition, and the importance of reuniting the disintegrated profession in this state, as well as the influence it will have on the medical affairs of the country.

It is high time the scandalous condition entailed by rival societies should cease.

No high-minded man, even to gratify personal ambition, will adopt obstructive tactics to even delay the prospective reuniting of these opposing and too long estranged forces. We hope the good sense of the leaders will enable them to reach a satisfactory conclusion promptly, and that the next meeting of the American

Medical Association, to be held at Saratoga, in June, 1902, will witness the welding of the links in the chain of professional brotherhood that were broken at St. Paul just twenty years before.

Since the foregoing was put into type the *Medical News* of December 21, has appeared with its second editorial article on the importance of reconciliation between the Society and the Association. The *News* take a comprehensive and liberal view of the subject in both articles, and in the latter says:

> It is evident, then, since at last there has come to both parties the realisation of the misconception that caused disunion that we may look for a speedy reconciliation. The Medical Society of the State of New York deserves the most respectful consideration if it shows a willingness to enter the fold of the National Association from which, by an unfortunate misunderstanding rather than any clash of principles, it has been so long excluded.

If the spirit manifested in the *News's* paragraph above quoted should characterise the Association conferees, the reunion is as good as accomplished.

